# Prognosis of remaining fetus in twin pregnancy after demise of one fetus according to its location

**DOI:** 10.1186/s12884-024-06621-w

**Published:** 2024-06-14

**Authors:** Young Mi Jung, Sun Min Park, Hyun Ji Kim, Bo Young Choi, Seunghyun Won, Jee Yoon Park, Kyung Joon Oh

**Affiliations:** 1 Department of Obstetrics and Gynecology, Korea University College of Medicine, Seoul, Republic of Korea; 2 Department of Obstetrics and Gynecology, Seoul National University Bundang Hospital, Seoul National University College of Medicine, 82 Gumi-ro, 173 beon-gil, Bundang-gu, Seongnam-si, Gyeonggi-do 13620 Republic of Korea; 3 Medical Research Collaborating Center, Seoul National University Bundang Hospital, Seongnam, Republic of Korea

**Keywords:** Fetal demise, Twin pregnancy, Abortion, Stillbirth, Preterm birth

## Abstract

**Background:**

To investigate the prognosis of the remaining fetus in twin pregnancy after experiencing one fetal demise in the first trimester according to the location of the demised fetus.

**Methods:**

This was a retrospective study of twin pregnancies with one fetal demise after the first trimester (14 weeks of gestation) delivered between September 2004 and September 2022. The study population was divided into two groups based on the location of the demised fetus as determined by the last recorded ultrasonography results: Group 1 included twin pregnancies where the presenting fetus was demised (*n* = 36) and Group 2 included twin pregnancies where the non-presenting fetus was demised (*n* = 44). The obstetric and neonatal outcomes were also reviewed.

**Results:**

A total of 80 pregnant women were included. The median gestational age for the diagnosis of fetal demise was 24.1 weeks. The gestational age of the demised fetus was not different between Groups 1 and 2; however, the gestational age of the remaining fetus at delivery was significantly earlier in Group 1 than it was in Group 2 (33.8 vs. 37.3 weeks, *P* = .004). The rate of preterm birth before 28 weeks was almost five times higher in Group 1 than in Group 2 (22.2% vs. 4.5%, *P* = .037). Regression analysis demonstrated significant differences between Groups 1 and 2. Respiratory distress syndrome, bronchopulmonary dysplasia, patent ductus arteriosus, retinopathy of prematurity, and jaundice were more common in Group 1 than in Group 2; however, the association was not significant after adjusting for gestational age at delivery.

**Conclusions:**

When the presenting fetus is demised in a twin pregnancy, the remaining fetus tends to be delivered earlier than when the non-presenting fetus is demised.

## Background

In multifetal pregnancies, the most common causes of fetal death are related to complications such as fetal anomalies and monochorionic-related issues including fetal growth restriction, twin-twin transfusion syndrome and twin anemia-polycythemia sequence [1]. When fetal death occurs early, there is no increased risk of death for the surviving fetus after the first trimester, and no need for additional surveillance to ensure survival.

However, the incidence of one fetal death in twin pregnancies after 20 weeks is 3.3–3.8% [2, 3]. Fetal death can have a significant impact on the health and survival of the remaining twin and the mother [4]. In cases of monochorionic twin pregnancies, the surviving twin may experience acute hypovolemia due to volume shifts within the placental anastomoses, leading to neurological injury or even the death of the surviving twin. Additionally, demise of one fetus could potentially trigger coagulation defects in the mother. However, while the risk of preterm birth increases after one fetal demise, there is no difference in the risk between dichorionic and monochorionic twin pregnancies [5]. 

The outcome of a pregnancy in which one twin has demised varies depending on several factors, including the gestational age at which death occurred, the cause of death, and the position of the demised twin. The demised twin may remain inside the uterus until delivery of the surviving twin, which, in some cases, can lead to complications such as infection, bleeding, coagulation abnormalities, or preterm labor. Previous studies on the location of the demised twin and its association with pregnancy outcomes are scarce, likely due to the low incidence of fetal death occurring after the first trimester. However, one study demonstrated that in twin pregnancy with one fetal demise, there may be worse outcomes for the pregnancy and survivors if the demised twin is presenting [1]. 

The objective of this study was to investigate the prognosis of the remaining fetus in twin pregnancy after experiencing one fetal demise after the first trimester and to compare the outcomes according to the location of the demised fetus.

## Materials and methods

### Study design

This retrospective cohort study included twin pregnancies with one fetal demise after the first trimester (14 weeks of gestation) that were delivered at the tertiary center between September 2004 and September 2022. We excluded multiple pregnancies of a higher order, cases in which both twins died, cases with unavailable or incomplete neonatal outcomes, cases in which fetal death was first diagnosed at delivery, and cases of pregnancy termination before 20 weeks. This study was approved by the Institutional Review Board of the center (no. B-1905/540-005) and followed the principles set forth in the Helsinki Declaration of 1975, as revised in 2013.

### Management strategies of twin pregnancy

The management strategies for twin pregnancies in our center are as follows: (1) In pregnancies conceived spontaneously, the larger of the two crown-rump lengths was used to estimate gestational age; (2) Chorionicity was determined before 14 weeks of gestation; (3) After one fetal death in monochorionic twins, the surviving twin was assessed for anemia, brain damage, and ongoing fetal compromise. If there was no evidence of anemia, brain damage, or ongoing fetal compromise, delivery was usually performed at 34–36 weeks of gestation; (4) In the case of fetal demise in a dichorionic twin, the well-being status of the surviving fetal was assessed to determine the appropriate management strategy.

### Baseline characteristics and pregnancy outcomes

We collected data on the following maternal characteristics and pregnancy outcomes: age, parity, mode of conception, height, weight, chorionicity, history of selective fetal reduction, gestational age at the fetal demise, diabetes, pregnancy complications (pregnancy-induced hypertension, gestational diabetes, placenta previa, oligohydramnios, preterm labor, and preterm premature rupture of membranes), history of cerclage, antenatal corticosteroid administration, gestational age at delivery, time interval from one fetal demise to the delivery of the remaining fetus, and mode of delivery. Chorioamnionitis and funisitis were defined based on histopathological evidence.

### Neonatal outcomes

The twin order was determined by reviewing the latest ultrasonographic results. A twin closer to the cervix at the time of the diagnosis of fetal death in utero was considered presenting. Neonatal outcomes including neonatal mortality and morbidity, such as respiratory distress syndrome (RDS), bronchopulmonary dysplasia (BPD), pulmonary hemorrhage, pneumonia, apnea, use of continuous positive airway pressure, use of mechanical ventilator support, patent ductus arteriosus (PDA), sepsis, hypoglycemia, disseminated intravascular coagulopathy (DIC), anemia, germinal matrix hemorrhage (GMH), intraventricular hemorrhage (IVH), periventricular leukomalacia (PVL), necrotizing enterocolitis (NEC), retinopathy of prematurity (ROP), jaundice, developmental delay, and cerebral palsy (CP) were reviewed. These outcomes were confirmed using electronic medical records.

Briefly, RDS was diagnosed as the presence of respiratory distress, an increased oxygen requirement (FiO_2_ > 0.4), and diagnostic radiological and laboratory findings in the absence of evidence of any other causes of respiratory distress [6]. The diagnosis of BPD was made using the definition criteria of the National Institute of Child Health Workshop, i.e., treatment with oxygen > 21% for at least 28 days, and also in the presence of typical findings at autopsy [7]. Congenital neonatal sepsis was diagnosed in the presence of a positive blood culture result within 72 h of delivery [8]. When abdominal distension and feeding intolerance (vomiting or increased gastric residual) for at least 24 h with evidence of intramural air, perforation, and meconium plug syndrome by radiological examination were found, NEC was confirmed through surgical exploration [9]. For brain lesions, IVH was diagnosed by ultrasonographic examination or magnetic resonance imaging (MRI) of the neonatal head (≥ Grade II) [10] and PVL was diagnosed as the presence of cystic lesions within the peri-ventricular white matter by ultrasonographic examination or MRI [11]. 

Neurodevelopmental assessment was performed using the Bayley scale at the pediatric rehabilitation clinic, followed by consultation with the attending physician, which yielded scores for mental and psychomotor development. Scores less than 85 were considered impaired or delayed developmental status [12]. The diagnosis of CP was made when the presence of definite abnormalities on the neurodevelopmental assessment (i.e., abnormalities of developmental milestones, posture evaluated by the Vojta method, and reflex) and persistent abnormalities of muscle tone were found [13]. 

### Statistical anlysis

Proportions were compared using Fisher’s exact test, and comparisons of continuous variables between groups were performed using the Mann–Whitney U test. The adjusted odds ratios (ORs) and 95% confidence intervals (CI) for the risk of adverse neonatal outcomes according to the position of the demised twin were calculated using multivariable logistic regression analysis. Gestational age at delivery was statistically different between the two groups, so this variable was adjusted. Given the assumption of unequal variances, the Generalized Wilcoxon test (also known as Brunner Munzel test) was used to compare the gestational age at delivery and the interval from one fetal demise to the delivery of the remaining fetus between the presenting twin-demised group (Group 1) and the non-presenting twin-demised group (Group 2). All statistical analyses were performed using SPSS version 29 for Windows (IBM Corp., Armonk, NY, USA). Statistical significance was set at a two-sided p-value < 0.05.

## Results

**Fig. 1:**
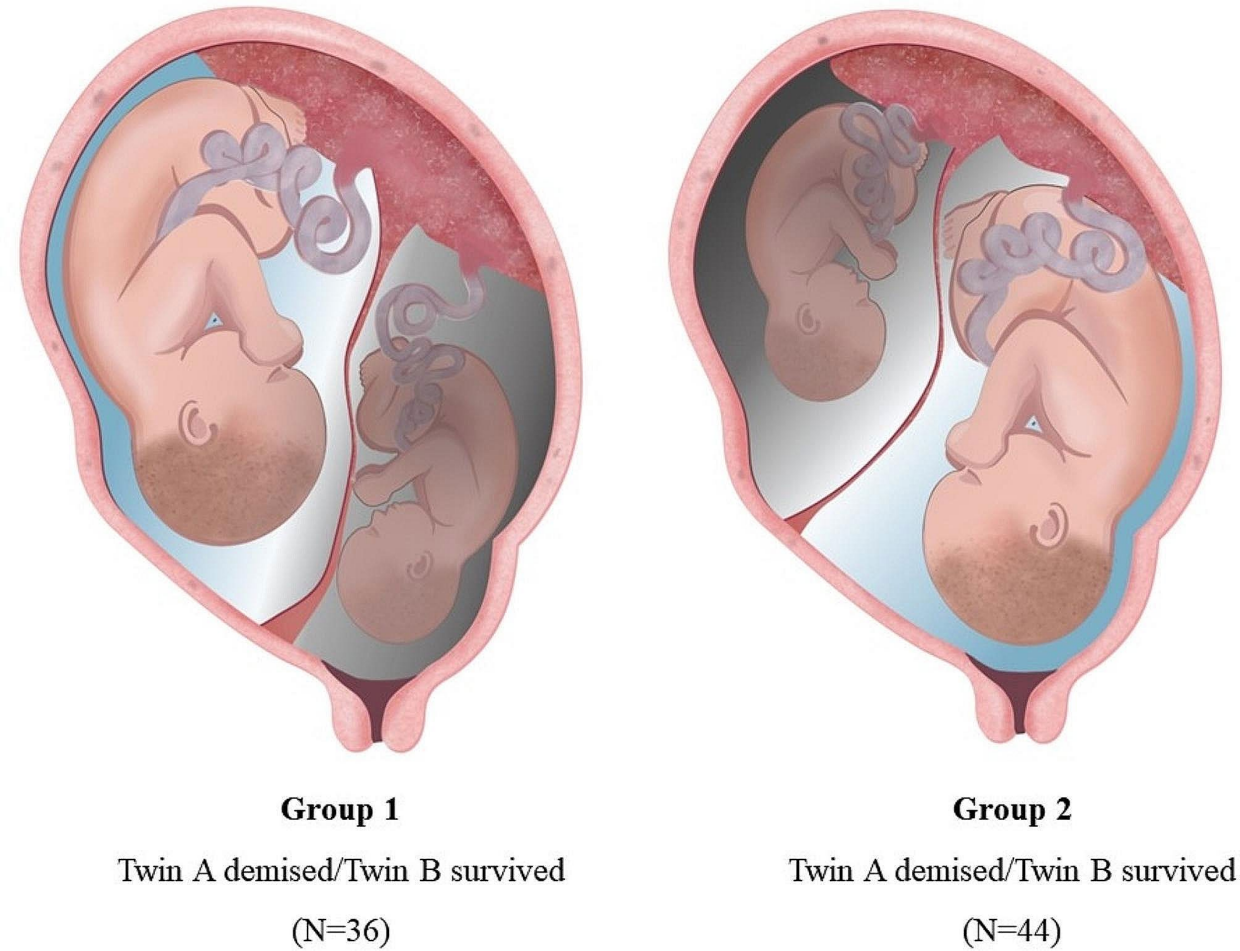
Flow chart of the study population The study population was divided into two groups: the demised presenting twin group (**Group 1**) and the demised non-presenting twin group (**Group 2**)

A total of 2,892 women pregnant with twins were admitted to our hospital during the study period. Except for seven pregnancies with a diagnosis of fetal demise between 10 + 0 and 13 + 6 weeks of gestation, 80 pregnancies that met the inclusion criteria were included in this study. The population was divided into two groups according to the position of the deceased fetus. Group 1 included twin pregnancies in which the presenting fetus was demised (*n* = 36), and Group 2 included twin pregnancies in which the non-presenting fetus was demised (*n* = 44), as determined by the last ultrasonography results (Fig. 1).

**Fig. 2:**
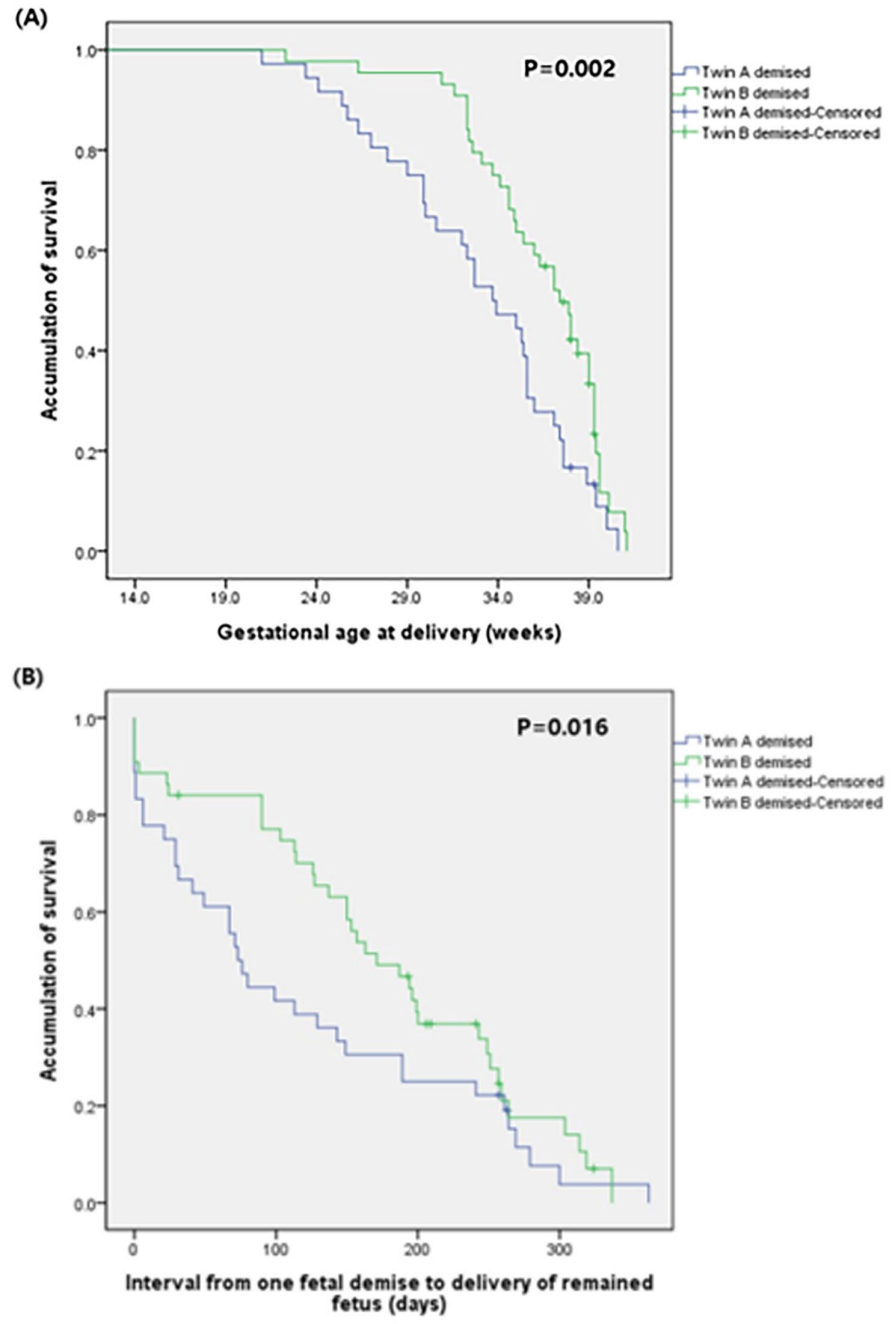
Survival analysis Survival analysis was performed using Generalized Wilcoxon test for (**A**) gestational age at delivery and (**B**) time interval from one fetal demise to delivery of remained fetus, comparing between the demised presenting twin group (Group 1) and the demised non-presenting twin group (Group 2)

Maternal age, parity, need for assisted reproductive techniques, pre-pregnancy body mass index (BMI), chorionicity, and the incidence of selective fetal reduction did not differ between the two groups. The median gestational age at which one fetal death occurred was 24.1 weeks in Group 1 and 24.3 weeks in Group 2. There were no significant differences in the rates of pregnancy complications such as pregnancy-induced hypertension, gestational or overt diabetes, placenta previa, oligohydramnios, preterm labor, preterm premature rupture of membranes, cerclage operation, histologic chorioamnionitis, and funisitis. However, the gestational age at delivery was significantly earlier in Group 1 than it was in Group 2 (33.8 vs. 37.3 weeks, *P* = .004), and the rate of preterm birth before 28 weeks was almost five times higher in Group 1 than in Group 2 (22.2% vs. 4.5%, *P* = .037). The cesarean section rate did not differ between the two groups. (Table 1) Survival analysis showed a significantly earlier gestational age at delivery in Group 1 than in Group 2 (*P* = .002) (Fig. 2A). In pregnancies with one fetal demise, there was a significantly shorter interval from one fetal demise to the delivery of remaining fetus compared in Group 1 than in Group 2 (*P* = .016) (Fig. 2B).

**Table 1:** Baseline characteristics and pregnancy outcomes compared between the groups

	Group 1 (*n* = 36)	Group 2 (*n* = 44)	*P*-value
Age (years)	34 (32–36)	34 (31–35)	0.690
Nulliparity	86.1% (31/36)	88.6% (39/44)	0.747
ART	52.8% (19/36)	61.4% (27/44)	0.499
Height (cm)	161.0 (158.0–165.0)	161.0 (157.5–165.3)	0.820
Weight at delivery (kg)	68.5 (60.6–72.7)	68.5 (63.8–77.5)	0.355
Pre-pregnancy weight (kg)	56.5 (52.0–62.2)	56.0 (48.0–62.0)	0.598
Pre-pregnancy BMI (kg/m^2^)	20.7 (19.6–23.6)	21.6 (19.2–24.0)	0.992
Monochorionic twin	22.2% (8/36)	11.4% (5/44)	0.232
Selective fetal reduction	13.9% (5/36)	22.7% (10/44)	0.394
Gestational age at one fetal demise (weeks)	24.1 (20.7–29.0)	24.3 (20.7–27.7)	0.835
Pregnancy outcomes			
Pregnancy induced hypertension	0% (0/36)	6.8% (3/44)	0.248
Gestational or Pregestational DM	11.1% (4/36)	11.4% (5/44)	> 0.999
Placenta previa	5.6% (2/36)	6.8% (3/44)	> 0.999
Oligohydramnios	2.8% (1/36)	9.1% (4/44)	0.372
PTL with tocolytics administration	30.6% (11/36)	11.4% (5/44)	0.049
PPROM	25.0% (9/36)	11.4% (5/44)	0.143
Cerclage operation	0% (0/36)	4.5% (2/44)	0.499
Antenatal corticosteroid administration	22.2% (8/36)	11.4% (5/44)	0.232
Gestational age at delivery (weeks)	33.8 (29.2–37.3)	37.3 (33.8–39.0)	0.004
Preterm birth < 37 weeks	72.2% (26/36)	45.5% (20/44)	0.023
Preterm birth < 34 weeks	52.8% (19/36)	25.0% (11/44)	0.020
Preterm birth < 32 weeks	36.1% (13/36)	9.1% (4/44)	0.005
Preterm birth < 28 weeks	22.2% (8/36)	4.5% (2/44)	0.037
Interval from one fetal demise to delivery of remained fetus (days)	75 (23–228)	167 (93–248)	0.041
Cesarean section	52.8% (19/36)	54.5% (24/44)	> 0.999
Acute histologic chorioamnionitis	36.4% (12/33)	27.5% (11/40)	0.456
Funisitis	6.1% (2/33)	7.5% (3/40)	> 0.999

ART: assisted reproductive technology; BMI: body mass index; DM, diabetes mellitus; PTL: preterm labor; PPROM: preterm premature rupture of membranes

Values are expressed as medians (interquartile ranges) for continuous variables and as percentages for categorical variables

Table 2 shows the neonatal outcome rates based on the location of the deceased fetus. Of the 80 surviving fetuses, three of them died immediately after delivery. The overall survival rate was 93.7% (75/80), and there was no statistically significant difference in the survival rates based on the location of the demised fetus. Additionally, fetal sex, rates of low Apgar scores, rates of meconium-stained amniotic fluid, rates of low cord pH, rates of congenital anomalies, and admission to the neonatal intensive care unit did not differ between the two groups. Due to the difference in gestational age at delivery, birth weight was lower in Group 1 than in Group 2 (1870 g vs. 2590 g, *P* = .005). However, the rate of small-for-gestational-age fetuses did not differ between the two groups.

**Table 2:** Neonatal outcomes compared between the groups

	Group 1 (*n* = 36)	Group 2 (*n* = 44)	*P*-value
Male baby	58.3% (21/36)	65.9% (29/44)	0.498
Birthweight (grams)	1870 (982–2728)	2590 (1865–3008)	0.005
Small for gestational age	11.8% (4/34)	7.0% (3/43)	0.693
Expired at delivery	5.6% (2/36)	2.3% (1/44)	0.585
Low Apgar score at 1 min < 7	55.6% (20/36)	34.1% (15/44)	0.071
Low Apgar score at 5 min < 7	19.4% (7/36)	15.9% (7/44)	0.771
Meconium staining	5.6% (2/36)	11.4% (5/44)	0.449
Umbilical cord pH < 7.2	20.6% (7/34)	13.5% (5/37)	0.532
Congenital anomaly	0% (0/36)	2.3% (1/44)	> 0.999
NICU admission	64.7% (22/34)	48.8% (21/43)	0.176
Hospital stay (days)	21 (5–52)	6 (3–24)	0.010
RDS	26.5% (9/34)	7.0% (3/43)	0.027
BPD	24.2% (8/33)	4.7% (2/43)	0.017
Pulmonary hemorrhage	8.8% (3/34)	0% (0/43)	0.082
Pneumonia	5.9% (2/34)	11.6% (5/43)	0.455
Apnea	38.2% (13/34)	20.9% (9/43)	0.129
CPAP	41.2% (14/34)	34.9% (15/43)	0.639
Mechanical ventilator support	35.3% (12/34)	16.3% (7/43)	0.067
PDA	32.4% (11/34)	7.0% (3/43)	0.006
Early-onset sepsis	14.7% (5/34)	2.3% (1/43)	0.082
Hypoglycemia	8.8% (3/34)	11.6% (5/43)	> 0.999
DIC	5.9% (2/34)	2.3% (1/43)	0.580
Anemia	14.7% (5/34)	2.3% (1/43)	0.082
GMH	11.8% (4/34)	14.0% (6/43)	> 0.999
IVH	5.9% (2/34)	2.3% (1/43)	0.580
ICH	2.9% (1/34)	0% (0/43)	0.442
PVL	2.9% (1/34)	0% (0/43)	0.442
NEC	11.8% (4/34)	2.3% (1/43)	0.164
ROP	18.2% (6/33)	0% (0/43)	0.005
Jaundice with phototherapy	52.9% (18/34)	23.3% (10/43)	0.009
Developmental delay	6.2% (2/32)	2.3% (1/43)	0.572
Overall survival	88.9% (32/36)	97.7% (43/44)	0.169

NICU: neonatal intensive care unit; RDS: respiratory distress syndrome; BPD: bronchopulmonary dysplasia; CPAP: continuous positive airway pressure; PDA: patent ductus arteriosus; DIC: disseminated intravascular coagulopathy; GMH: germinal matrix hemorrhage; IVH: intraventricular hemorrhage; ICH: intracranial hemorrhage; PVL: periventricular leukomalacia; NEC: necrotizing enterocolitis; ROP: retinopathy of prematurity

Values are expressed as medians (interquartile ranges) for continuous variables and as percentages for categorical variables

The risk of adverse neonatal outcomes was compared between the two groups, and it was found that some outcomes such as RDS, BPD, PDA, ROP, and jaundice were more prevalent in Group 1 than in Group 2. However, after adjusting for the gestational age at delivery, there was no significant differences in the odds ratio of these outcomes between the two groups (Table 3).

**Table 3:** Odds ratio of association between demise of presenting fetus in twin pregnancy and adverse neonatal outcomes

	OR (95% CI)	aOR (95% CI)	*P*-value*
RDS	4.80 (1.19–19.44)	1.18 (0.17–8.08)	0.864
BPD	6.56 (1.23–33.39)	2.35 (0.32–17.37)	0.401
Pulmonary hemorrhage	NA	NA	NA
Pneumonia	0.48 (0.09–2.62)	0.13 (0.01–1.23)	0.075
Apnea	2.34 (0.85–6.42)	1.02 (0.30–3.47)	0.971
CPAP	1.31 (0.52–3.30)	0.42 (0.12–1.47)	0.174
Mechanical ventilator support	2.81 (0.96–8.20)	0.83 (0.19–3.59)	0.798
PDA	6.38 (1.61–25.24)	2.66 (0.52–13.71)	0.241
Early-onset sepsis	7.24 (0.80–65.26)	3.95 (0.38–40.93)	0.249
Hypoglycemia	0.74 (0.16–3.32)	0.51 (0.10–2.78)	0.440
DIC	2.63 (0.23–30.24)	0.63 (0.03–12.41)	0.763
Anemia	7.27 (0.80–65.26)	4.05 (0.39–41.83)	0.240
GMH	0.82 (0.21–3.18)	0.25 (0.04–1.51)	0.131
IVH	2.63 (0.23–30.24)	0.51 (0.02–11.00)	0.668
ICH	NA	NA	NA
PVL	NA	NA	NA
NEC	5.60 (0.60–52.65)	2.78 (0.25–31.50)	0.409
ROP	NA	NA	NA
Jaundice with phototherapy	3.71 (1.40–9.86)	2.63 (0.93–7.46)	0.068
Developmental delay	2.80 (0.24–32.31)	0.68 (0.04–13.21)	0.409
Overall survival	0.19 (0.02–1.75)	0.41 (0.01–36.19)	0.699

*Adjusted for gestational age at delivery

OR: odds ratio; RDS: respiratory distress syndrome; BPD: bronchopulmonary dysplasia; NA: not available; CPAP: continuous positive airway pressure; PDA: patent ductus arteriosus; DIC: disseminated intravascular coagulopathy; GMH: germinal matrix hemorrhage; IVH: intraventricular hemorrhage; ICH: intracranial hemorrhage; PVL: periventricular leukomalacia; NEC: necrotizing enterocolitis; ROP: retinopathy of prematurity

## Discussion

This study’s main finding was that the gestational age of the remaining fetus at delivery was significantly earlier when the presenting fetus was deceased. Additionally, the rate of preterm births before 28 weeks of gestation was significantly higher when the presenting fetus was demised. Furthermore, the rates of a number of neonatal outcomes, including RDS, BPD, PDA, ROP, and jaundice, were higher in the demised presenting fetus group, but the association was not significant after adjusting for gestational age at delivery.

A previous study on co-twin prognosis after spontaneous single intrauterine fetal death found that, in monochorionic twins, if death occurred before 28 weeks of gestation, there was a higher rate of co-twin intrauterine and neonatal deaths. Neonatal death was more likely in the presence of fetal growth restriction or preterm birth. Surviving monochorionic co-twins had abnormal antenatal brain imaging in 20% of the cases [14]. Penelope et al. found that, if a demised twin was present, delivery occurred earlier and resulted in more neonatal morbidity for the surviving twin; however, the position of the demised twin did not affect neonatal morbidity after adjusting for gestational age. The study also found a higher rate of anomalies in surviving twins when the presenting twin was deceased. Maternal morbidity was mostly unrelated to the position of the demised twin, except for emergency cesarean delivery, which was more common when the presenting fetus had died [1]. Consistently, in the current study, we found that non-respiratory morbidities, such as PDA, ROP, and jaundice, increase in cases where the presenting fetus is demised. These morbidities did not differ between the two groups after adjusting for gestational age.

The differences in neonatal outcomes seem to be mainly attributable to prematurity. Moreover, covariates that may affect neonatal outcomes (method of conception, chorionicity, maternal BMI, rate of selective fetal reduction, gestational age at the fetal demise, underlying maternal disease, rate of oligohydramnios, rate of rupture of membranes, rate of antenatal steroid administration, and rate of intraamniotic infection) and others did not differ between the groups. Therefore, we could analyze the possible effects of the position of the demised fetus and gestational age at delivery on neonatal morbidity with minimal confounding factors.

In cases where the presenting fetus had died, there may have been a greater likelihood of the deceased fetus becoming a source of infection, which could have led to earlier delivery. In this study, we observed that when the presenting fetus had deceased, the median gestational age at delivery was approximately three weeks earlier. Additionally, although not statistically significant, we noted higher rates of premature rupture of membranes and intraamniotic infections.

Previous studies on the effect of birth order and neonatal morbidity in twin pregnancies have shown that the rate of mortality was lower in the non-presenting babies; however, the rates of RDS and ROP were higher in the non-presenting babies. The higher risk of mortality in presenting twins may be related to closer contact with the infected environment and a higher rate of intraamniotic infection [15, 16]. One possible explanation for the increased incidence of pulmonary complications among second-born babies is that they typically do not initiate labor, and therefore do not receive natural corticosteroids that promote lung maturation as often as first-born twins. Consequently, the respiratory system may have been less developed and more susceptible to complications [17]. 

Thus, it is possible that inflammation and infection resulting from the demise of the presenting fetus may have been further accelerated by exposure to the external environment through the cervix, leading to an increased risk of preterm labor and subsequent preterm births. After a fetal death, the decision regarding the mode of delivery is generally less restricted than that in a twin pregnancy. In this study, since there was no significant difference in the rates of cesarean sections between the groups, the influence of labor-related stress on neonatal morbidity was minimal, and much of the morbidity was attributable to prematurity.

Fetal death after 20 weeks of gestation has a very low incidence of 3–4%. One strength of our study is that our hospital is a referral center for twin pregnancies, allowing us to investigate this rare incidence. Moreover, this is the very unique study to systematically confirm important neonatal morbidities through the direct examination of medical records. However, owing to the retrospective nature of this study, we were unable to evaluate the exact reasons for fetal demise. Second, although chorionicity did not differ between groups, the sample size was too small to conduct a subgroup analysis based on chorionicity. Third, the limitations of this study include also a relatively small sample size and an extended study duration, which might influence the robustness and applicability of the findings. Additionally, changes in pediatric neonatal care capacity during the study period could introduce biases in the comparisons between different groups. These factors may affect the generalizability of the results and should be carefully considered when interpreting the findings. We acknowledge these potential constraints and suggest that future research should address these issues to validate and extend our conclusions.

## Conclusions

In conclusion, when the presenting fetus is demised in a twin pregnancy, the remaining fetus tends to be delivered earlier than when the non-presenting fetus is demised. Our findings may help identify cases with a higher risk of neonatal morbidity due to premature delivery, and could also be helpful in counselling patients facing the grief of one fetal demise.

## Data Availability

The datasets used and/or analysed during the current study are available from the corresponding author on reasonable request.

## Abbreviations

BMI: body mass index
BPD: bronchopulmonary dysplasia
CI: confidence intervals
CP: cerebral palsy
DIC: disseminated intravascular coagulopathy
GMH: germinal matrix hemorrhage
IVH: intraventricular hemorrhage
MRI: magnetic resonance imaging
NEC: necrotizing enterocolitis
PDA: patent ductus arteriosus
PVL: periventricular leukomalacia
OR: odds ratios
RDS: respiratory distress syndrome
ROP: retinopathy of prematurity
